# The Autism Simplex Collection: an international, expertly phenotyped autism sample for genetic and phenotypic analyses

**DOI:** 10.1186/2040-2392-5-34

**Published:** 2014-05-20

**Authors:** Joseph D Buxbaum, Nadia Bolshakova, Jessica M Brownfeld, Richard JL Anney, Patrick Bender, Raphael Bernier, Edwin H Cook, Hilary Coon, Michael Cuccaro, Christine M Freitag, Joachim Hallmayer, Daniel Geschwind, Sabine M Klauck, John I Nurnberger, Guiomar Oliveira, Dalila Pinto, Fritz Poustka, Stephen W Scherer, Andy Shih, James S Sutcliffe, Peter Szatmari, Astrid M Vicente, Veronica Vieland, Louise Gallagher

**Affiliations:** 1 The Seaver Autism Center for Research and Treatment, Department of Psychiatry, Icahn School of Medicine at Mount Sinai, New York 10029, USA; 2 Autism Genetics Group, Department of Psychiatry, School of Medicine, Trinity College, Dublin 8, Ireland; 3 National Institute of Mental Health (NIMH), Bethesda, MD 20892-9663, USA; 4 Department of Psychiatry and Behavioral Sciences, University of Washington, Seattle, WA 98195, USA; 5 Institute for Juvenile Research, Department of Psychiatry, University of Illinois at Chicago, Chicago, IL 60608, USA; 6 Psychiatry Department, University of Utah Medical School, Salt Lake City, UT 84108, USA; 7 The John P Hussman Institute for Human Genomics, University of Miami, Miami, FL 33101, USA; 8 Department of Child and Adolescent Psychiatry, Psychosomatics and Psychotherapy, JW Goethe University Frankfurt, 60528 Frankfurt, Germany; 9 Department of Psychiatry and Behavioral Science, Child and Adolescent Psychiatry, Stanford School of Medicine, Stanford, CA, USA; 10 Department of Neurology, University of California at Los Angeles, School of Medicine, Los Angeles, CA 90095, USA; 11 German Cancer Research Center (DKFZ), 69120 Heidelberg, Germany; 12 Department of Psychiatry, Indiana University School of Medicine, Indianapolis, IN 46202, USA; 13 Unidade de Neurodesenvolvimento e Autismo do Serviço do Centro de Desenvolvimento da Criança and Centro de Investigação e Formação Clinica, Pediatric Hospital, Centro Hospitalar e Universitário de Coimbra, 3000-602 Coimbra, Portugal; 14 University Clinic of Pediatrics and Institute for Biomedical Imaging and Life Science, Faculty of Medicine, University of Coimbra, 3000-602 Coimbra, Portugal; 15 Department of Molecular Genetics, The Centre for Applied Genomics, Hospital for Sick Children and McLaughlin Centre and University of Toronto, Toronto, ON, Canada; 16 Autism Speaks, New York, NY 10016, USA; 17 Department of Molecular Physiology and Biophysics, Vanderbilt Kennedy Center, Vanderbilt University, Nashville, TN 37232, USA; 18 Centers for Human Genetics Research and Molecular Neuroscience, Vanderbilt University, Nashville, TN 37232, USA; 19 Department of Psychiatry and Behavioural Neurosciences, McMaster University, Hamilton, ON L8N 3Z5, Canada; 20 Instituto Nacional de Saúde Dr Ricardo Jorge, 1649-016 Lisbon, Portugal; 21 Instituto Gulbenkian de Ciência, P-2781-901 Oeiras, Portugal; 22 BioFIG-Center for Biodiversity, Functional & Integrative Genomics, Campus da FCUL, C2.2.12, Campo Grande, 1749-016 Lisboa, Portugal; 23 The Research Institute at Nationwide Children’s Hospital, The Ohio State University, Columbus, OH, USA

## Abstract

**Background:**

There is an urgent need for expanding and enhancing autism spectrum disorder (ASD) samples, in order to better understand causes of ASD.

**Methods:**

In a unique public-private partnership, 13 sites with extensive experience in both the assessment and diagnosis of ASD embarked on an ambitious, 2-year program to collect samples for genetic and phenotypic research and begin analyses on these samples. The program was called The Autism Simplex Collection (TASC). TASC sample collection began in 2008 and was completed in 2010, and included nine sites from North America and four sites from Western Europe, as well as a centralized Data Coordinating Center.

**Results:**

Over 1,700 trios are part of this collection, with DNA from transformed cells now available through the National Institute of Mental Health (NIMH). Autism Diagnostic Interview-Revised (ADI-R) and Autism Diagnostic Observation Schedule-Generic (ADOS-G) measures are available for all probands, as are standardized IQ measures, Vineland Adaptive Behavioral Scales (VABS), the Social Responsiveness Scale (SRS), Peabody Picture Vocabulary Test (PPVT), and physical measures (height, weight, and head circumference). At almost every site, additional phenotypic measures were collected, including the Broad Autism Phenotype Questionnaire (BAPQ) and Repetitive Behavior Scale-Revised (RBS-R), as well as the non-word repetition scale, Communication Checklist (Children’s or Adult), and Aberrant Behavior Checklist (ABC). Moreover, for nearly 1,000 trios, the Autism Genome Project Consortium (AGP) has carried out Illumina 1 M SNP genotyping and called copy number variation (CNV) in the samples, with data being made available through the National Institutes of Health (NIH). Whole exome sequencing (WES) has been carried out in over 500 probands, together with ancestry matched controls, and this data is also available through the NIH. Additional WES is being carried out by the Autism Sequencing Consortium (ASC), where the focus is on sequencing complete trios. ASC sequencing for the first 1,000 samples (all from whole-blood DNA) is complete and data will be released in 2014. Data is being made available through NIH databases (database of Genotypes and Phenotypes (dbGaP) and National Database for Autism Research (NDAR)) with DNA released in Dist 11.0. Primary funding for the collection, genotyping, sequencing and distribution of TASC samples was provided by Autism Speaks and the NIH, including the National Institute of Mental Health (NIMH) and the National Human Genetics Research Institute (NHGRI).

**Conclusions:**

TASC represents an important sample set that leverages expert sites. Similar approaches, leveraging expert sites and ongoing studies, represent an important path towards further enhancing available ASD samples.

## Background

Autism spectrum disorder (ASD) is a highly heritable neurodevelopmental disorder characterized by fundamental deficits in social reciprocity and the presence of restricted interests and/or repetitive behaviors [1]. ASD is among the most prevalent of developmental disorders with an estimated prevalence of about 1 in 100, with males affected at a rate four times that of females [2]. A substantial portion of the risk for ASD traces to genetic variation, either inherited [1,3,4] or arising *de novo*[5-11]. Through the strong support of families, advocacy groups and international research initiatives, large collaborative networks have been formed to advance knowledge of genes associated with ASD, and in recent years, these efforts have led to dramatic progress in the understanding of the genetics of ASD. Recent findings identify examples of specific etiological mechanisms, while underscoring the highly complex nature of the genetic architecture in ASD. To date, a specific genetic etiology can be identified in up to 25% of individuals with ASD and include single gene disorders (for example, Fragile X), known genetic syndromes (for example, 22q11 deletion syndrome), chromosomal anomalies, *de novo* and inherited copy number variation (CNV), indels, and single nucleotide variation (SNV) [12]. Intensive behavioral interventions can have profoundly positive effects on the prognosis of autism (for example, see [13]), however morbidity and the cost of ASD to society, families and individuals remains high [14,15], reinforcing the need for earlier diagnosis and ultimately, targeted treatment.

Further advances in the genetics of ASD will require analysis of genetic variation at all levels, necessitating the need to take advantage of new technology, including that of massively parallel (high-throughput) sequencing, and will require many thousands of samples to have adequate power to identify rare variants associated with high risk and more common variants associated with lower risk. The Interagency Autism Coordinating Committee (IACC) of the NIH, in its 2010 strategic plan, has called for genetic analysis of some 20,000 unrelated families, a number that is consistent with studies in other psychiatric disorders such as schizophrenia (which also has a similar prevalence and heritability to autism, see [16]). The IACC recognized that in order to discover additional causes of ASD to inform ‘prognosis and treatments and lead to the prevention/preemption of the challenges and disabilities of ASD’, the community would require such sample sizes.

In spite of the prevalence and burden of ASD, there have not yet been sufficient large-scale initiatives to recruit subjects to reach 20,000 unrelated families, and the numbers of unrelated families available to researchers through the National Institute of Mental Health (NIMH) Center for Collaborative Genetic Studies on Mental Disorders (CCGSMD) was still moderate in 2008. To begin to redress this, members of the Autism Genome Project Consortium (AGP) made a proposal to Autism Speaks to collect a large number of well-phenotyped trios and to make them publically available through the existing open process of the NIMH CCGSMD. The proposal attempted to be modest in terms of costs because all participating sites were already fully qualified on the relevant instruments, had families in clinical and research studies to turn to for participation, and all sites agreed to leverage ongoing studies funded by parallel sources to carry out the proposed sample collection at a fraction of the real costs. After positive review at Autism Speaks, and a critical parallel commitment by the NIMH to collect, transform, and distribute biomaterials and data through CCGSMD, The Autism Simplex Collection (TASC) was awarded funds to ascertain 1,700 trios in this initiative. As of today, the TASC samples are now available for qualified researchers through the NIMH CCGSMD and additional genotyping and whole exome sequencing (WES) data is being made available for these samples on an ongoing basis.

### The Autism Genome Project Consortium

In 2004, through the support of the National Alliance for Autism Research (NAAR), now Autism Speaks, the AGP was launched as an international consortium of genetic researchers from North America and Europe dedicated to identifying genes that underlie susceptibility to ASD. The goal of the AGP was to identify causes of ASD using modern genetic approaches and to then translate these findings into better clinical practice. The members of the AGP have joined together to reach these goals, recognizing that an open collaborative structure would allow the field to proceed with maximal efficiency. While a detailed description of the AGP is beyond the scope of the current manuscript, it is important to highlight some recent successes as they inter-relate with the goals of the TASC. The AGP has consistently made use of state-of-the-art methods, describing for the first time a genome-wide assessment of CNV in ASD that included a first report of CNV at the *NRXN1* gene in ASD [7]. The AGP reported a large genome-wide association study (GWAS) in ASD [17] and has identified many CNVs that contribute to ASD risk [6]. This latter study showed that CNV in genes previously associated with intellectual disability (ID) are likely contributing to ASD risk and identified pathways that appear to be implicated in ASD risk. The AGP collaborates with other initiatives, carrying out genotyping where requested (for example, see [18]) and providing all genotype data to the Psychiatric Genetics Consortium (PGC; [19]) for mega-analysis. Recently the AGP has completed genotyping another large sample (including much of TASC; Pinto *et al*. in press) and has contributed over 1,000 TASC samples for WES. WES data from cases was generated and analyzed, in a large effort funded by the NIMH and the National Human Genetics Research Institute (NHGRI), through an American Recovery and Reinvestment Act grant [3,8,20,21]. WES data from trios is now being generated by the ASC [22], again with support from the NIMH and NHGRI.

The AGP thus represents a unique union of organizations and research sites (over 50) that is comprised of clinicians and researchers with recognized expertise in ASD that work closely with local ASD groups and families. Since 2003, AGP members have published several hundred peer-reviewed papers on autism genetics research. The pooling of the extensive scientific and clinical expertise from across the world can serve to provide reliably characterized samples of phenotype and genotype data to the scientific community. This is critical as large, reliable samples are crucial for dissection of the genetic and genomic architecture of ASD.

### The Autism Simplex Collection (TASC)

In 2008, the AGP sought to provide an independent ASD sample for gene discovery and phenotypic analyses, and for replicating and extending findings from prior studies carried out by the AGP and by additional research groups. To this end, 13 sites within the AGP with extensive experience in the diagnosis and assessment of ASD made a proposal that included recruitment of additional families to the NIMH CCGSMD in a two-year period. Recruitment would include in-depth expert phenotyping of all families and biomaterials from all families.

## Methods

Autism Speaks agreed to fund the collection of 1,700 samples, while the NIH agreed to assume the costs for collecting, transforming and distributing the samples, as well as warehousing all clinical information. Dr. Louise Gallagher coordinated the recruitment with principle investigators at each site taking local responsibility (see Table 1). Inclusion criteria included individuals aged between 3 and 21 years with a research diagnosis of autism/ASD based on the Autism Diagnostic Interview-Revised (ADI-R) and the Autism Diagnostic Observation Schedule (ADOS) (administered by research reliable raters). Individual IQs were preferably greater than or equal to 35 based on standardized testing and a full-scale or nonverbal mental age of 18 months or greater. Only full parent-child trios were included and the availability of an unaffected sibling was not a requirement for inclusion. Individuals with known medical or genetic causes of autism or a history consistent with Childhood Disintegrative Disorder were excluded. Other exclusion criteria included: extreme prematurity (< 1,000 grams or less than 32 weeks); prematurity with associated neurological complications; birth trauma with associated early neo-natal complications; significant brain injury; *in utero* exposure to medication known to be associated with autism, for example. retinoic acid, sodium valproate. The Phenotype Protocol was developed and agreed by the members of the AGP Phenotype Analytic Committee (Chair: Dr. Peter Szatmari). Additionally, consultation with the Simon Simplex Collection, which was also in a phase of phenotype protocol development, allowed for cross-comparability of some instruments. Core assessments carried out at all sites included ADI-R [23] and ADOS-G [24] for the probands, as well as standardized IQ measures, Vineland Adaptive Behavioral Scales (VABS) [25], Social Responsiveness Scale (SRS) [26], Peabody Picture Vocabulary Test (PPVT) [27] and physical measures (height, weight, and head circumference) (Table 2). In addition, at almost every site, additional phenotype measures were assessed, including the Broad Autism Phenotype Questionnaire (BAPQ) [28] and Repetitive Behavior Scale-Revised (RBS-R) [29], as well as the non-word repetition scale (from the Comprehensive Test of Phonological Processing/CTOPP-Domain V) [30], The Children’s Communication Checklist-2 [31], Communication Checklist-Adult Version [32], and Aberrant Behavior Checklist (ABC) [33] (Table 2). Phenotypes were uploaded to a centralized database in the Data Coordinating Center (DCC), which performed point-of-entry data validation, facilitated cross-site harmonization of clinical information, and provided combined output data for analysis to ensure a common starting point for analyses performed at different participating sites.

**Table 1:** Target The Autism Simplex Collection (TASC) sample contribution by site

**Site**	**Year 1**	**Year 2**	**Total number of trios**
Portugal (AM Vicente and G Oliveira, site co-PIs)	183	97	280
Mount Sinai (JD Buxbaum, site PI)	125	106	231
Ireland (L Gallagher, TASC PI and site PI)	100	102	202
Canada (P Szatmari, site PI)	100	85	185
Utah (H Coon, site PI)	100	85	185
Miami (M Cuccaro, site PI)	100	76	176
Vanderbilt Univ (JS Sutcliffe, site PI)	70	59	129
Frankfurt (CM Freitag and F Poustka site co-PIs) and Heidelberg (SM Klauck, site PI)	59	50	109
AGRE (D Geschwind, site PI)	50	42	92
Indiana (JI Nurnberger, site PI)	40	34	74
Chicago (EH Cook, site PI)	20	30	50
CPEA (R Bernier, site PI)	5	4	9
Total			1,719

**Table 2:** Phenotype measures in The Autism Simplex Collection (TASC) samples

**Additional Measures**	**AGRE**	**Canada**	**Chicago**	**Washington**	**Germany**	**Ireland**	**Miami**	**Portugal**	**Utah**	**Vanderbilt**	**Mount Sinai**	**Indiana**
ADI	Yes	Yes	Yes	Yes	Yes	Yes	Yes	Yes	Yes	Yes	Yes	Yes
ADOS	Yes	Yes	Yes	Yes	Yes	Yes	Yes	Yes	Yes	Yes	Yes	Yes
Peabody Picture Vocabulary Test (PPVT)	Yes	Yes	Yes	Yes	Yes	Yes	Yes	Yes	Yes	Yes	Yes	Yes
Nonword repetition scale	No	Yes	Yes	Yes	No	Yes	Yes	Yes	Yes	Yes	Yes	Yes
Children’s communication checklist-2	No	Yes	Yes	No	CCC-1	Yes	No	Yes	Yes	Yes	Yes	Yes
Communication checklist-adult	No	Yes	Yes	No	No	Yes	No	Yes	Yes	Yes	Yes	Yes
Standardized measure of IQ	Yes	Yes	Yes	Yes	Yes	Yes	Yes	Yes	Yes	Yes	Yes	Yes
Vineland adaptive behavior scales	Yes	Yes	Yes	Yes	Yes	Yes	Yes	Yes	Yes	Yes	Yes	Yes
Social responsiveness scale	Yes	Yes	Yes	Yes	Yes	Yes	Yes	Yes	Yes	Yes	Yes	Yes
BAPQ	No	Yes	Yes	Yes	No	Yes	No	Yes	Yes	Yes	Yes	Yes
Physical measures (height, weight, head circumference)	Yes	Yes	Yes	Yes	Yes	Yes	Yes	Yes	Yes	Yes	Yes	Yes
Repetitive behavior scale-revised	Yes	Yes	Yes	Yes	No	Yes	Yes	Yes	Yes	Yes	Yes	Yes
Aberrant behavior checklist	No	Yes	Yes	Yes	Yes	Yes	Yes	Yes	Yes	Yes	Yes	Yes

CCC-1, Children’s Communication Checklist-1.

## Results

The consortium recruited over 90% of its target sample in approximately two and a half years. The ambitious recruitment was made feasible by capitalizing on sites with active clinical and research programs, already utilizing standardized, research-quality evaluation methods, and by leveraging site-specific resources. The TASC collection includes a majority of European ancestry, but also includes samples of more diverse ancestry. Summary statistics on ancestry, as well as ADI-R and ADOS-G diagnoses, and available genetic data are shown Table 3.

**Table 3:** Summary data The Autism Simplex Collection (TASC) sample

	**N**	**%**
**Total sample**		
**Individuals**	5444	
**Families**	1719	
**Children**	1997	
Male children	1643	82.4
Female children	354	17.8
**Genetic data available**		
CNV data	2666	49.0
GWA data	2934	53.9
Whole exome data (not including ongoing ASC data)	530	29.4 (cases only)
**GWA-derived ancestry**		
European	2443	83.3
African	66	2.2
Latino	352	12.0
Asian	73	2.5
**Classification**		
ADI (ASD1 or ASD2 [34] or autism)	1464	
ADI (autism) (subset of above counts)	1339	91.5
ADOS (ASD or autism)	1262	
ADOS (autism) (subset of above counts)	1008	79.9

## Discussion

### Genetic analyses of TASC samples

AGP members continue their work on ASD genetics and have been making use of TASC samples to this end. Over 400 TASC families are included in the two recent AGP papers reporting on CNV and common SNPs in ASD [6,17]. Additional TASC families are in the current analysis in the follow-up AGP cohort (Pinto *et al*., in press), such that to date, both genome-wide SNP data and CNV calls have been generated for some 900 TASC families. This data is being analyzed by the AGP and shared with other sites, including the PGC [19] as well as the database of Genotypes and Phenotypes (dbGaP) and the National Database for Autism Research (NDAR). Genotypes were managed and stored centrally in the DCC along with phenotypes and were transferred from there to the national databases. Called CNV are also available for TASC samples.

The TASC samples were also an important part of studies sequencing over 1,000 ASD exomes using next-generation sequencing. Five sites collaborated on this American Recovery and Reinvestment Act (ARRA)-funded initiative, where a premium was placed on making use of publically available samples that could be matched with control samples using genome-wide SNP data. Using genome-wide SNP data generated by the AGP from the approximately 950 TASC families, over 500 unrelated TASC ASD subjects were selected for WES [3,8,20,21]. Sequencing data from these families were released early 2012 via dbGaP. The same ARRA grant provided resources to facilitate the release of TASC samples to additional researchers through the NIMH CCGSMD. Most recently, the ASC [22] has begun to sequence whole blood-derived DNA from TASC samples, with support from the NIMH and NHGRI. WES data from 1,000 samples (primarily in the form of trios) will be released through dbGaP in 2014, and data from additional TASC samples will be released as the data become available.

### TASC in the broader context

The goal identified by the IACC to examine 20,000 ASD probands for genetic factors is consistent with studies in other complex psychiatric disorders. As noted above, both prevalence and heritability of schizophrenia are in the same range as is seen for ASD [16]. In addition, like ASD, schizophrenia risk includes CNV as a source of the risk architecture [35,36]. The PGC has published a GWAS with approximately 18,000 schizophrenia samples and identified over 10 SNPs with very modest effect sizes [37], with a larger study (with twice the number of affecteds) now being completed. The PGC study in ASD, which includes all available SNP data in ASD (and virtually all AGP and TASC data) accounts for over 5,000 samples. Clearly it is not yet possible to identify multiple common SNPs with very low effect sizes are part of the ASD risk, as it is clear that common SNPs with odds ratios greater than 1.3 can be excluded [38]. Even considering rare variation with major effect, large sample sizes are needed, precisely because the variants are rare. In short, there is every reason to think that the target of 20,000 proposed by the IACC is required and that this sample size might begin to achieve the associated goal crucial of the IACC, which is to capture a significant proportion of the genetic risk for ASD.

TASC shares similarities with other large-scale sample collection. These include the Autism Genetic Resource Exchange (AGRE) [39], a network originally funded by Cure Autism Now (CAN) and now integrated into Autism Speaks, and the Simons Simplex Collection [40], funded by the Simons Foundation (see Table 4). In addition, the NIMH has samples from prior collections, and efforts are being made to collect additional samples. Collectively, these important efforts can provide samples that approach those in schizophrenia over the next several years and provide greater power for broader analyses that will be required to fully comprehend the relationship between genotype and phenotype.

**Table 4:** Summary of available sample sets

**Sample set**	**Approximate number of unrelated samples**	**Design**	**Website**
TASC	1,700 trios	Simplex (no exclusion for additional ASD in the family	
AGRE	1,700 families	Primarily multiplex	http://www.agre.org
Simons Simplex Collection (SSC)	2,500 quads	Simplex with unaffected sib and no additional ASD in first degree relatives	https://sfari.org/resources/simons-simplex-collection
NIMH CCGSMD	15,000 samples in current release (with overlap with AGRE and TASC)	Multiple sources and designs	https://www.nimhgenetics.org/

## Conclusions

There has been remarkable progress in ASD genetics in the past several years, driven to no small degree by the availability of large well-characterized samples. Relevant genetic findings can now be made in over 25% of ASD cases, using Fragile X testing, chromosome microarray (CMA) and WES. Fragile X testing and CMA can identify genetic findings in more than 10% of cases. With WES, studies in the past two years have shown that deleterious *de novo*, recessive and X-linked mutations are observed in some 12% of subjects, and WES identifies small contributory CNV in some 7% of subjects [3,4,8-11,41,42]. Moreover, recently identified mutations and CNVs that are associated with high risk for ASD are leading to a better understanding of ASD pathogenesis and to model systems for ASD. Identification of high-risk genetic variants can provide an etiological diagnosis and opportunities for genetic counseling, an emerging important aspect in ASD care [43]. In some examples of monogenic forms of ASD, including Fragile X syndrome and tuberous sclerosis, model systems have led clinical trials with novel therapeutics, highlighting the translational nature of gene discovery in ASD research. Explaining a large proportion of the genetic risk for ASD over the next five years is an achievable goal, but will require larger collections of well-characterized samples. The TASC research group and the TASC samples represent a successful collaboration and public-private partnership to enhance the numbers of samples available through the NIMH in less than three years. The phenotypic and genetic data generated by the AGP and others provide unique high value to this collection. Furthermore, the use of these samples in many ongoing studies will continue to add value to this collection. Similar approaches, leveraging expert sites and ongoing studies, represent an important path towards further enhancing the available ASD sample set.

## Abbreviations

ABC: Aberrant Behavior Checklist; ADI-R: Autism Diagnostic Interview-Revised; ADOS: Autism Diagnostic Observation Schedule; ADOS-G: Autism Diagnostic Observation Schedule-Generic; AGP: Autism Genome Project Consortium; AGRE: Autism Genetic Research Exchange; ARRA: American Recovery and Reinvestment Act; ASC: Autism Sequencing Consortium; ASD: autism spectrum disorder; BAPQ: Broad Autism Phenotype Questionnaire; CAN: Cure Autism Now; CCGSMD: Center for Collaborative Genetic Studies on Mental Disorders; CMA: chromosome microarray; CNV: copy number variation; CTOPP: Comprehensive Test of Phonological Processing; dbGaP: Database of Genotypes and Phenotypes; GWAS: genome-wide association study; IACC: Interagency Autism Coordinating Committee; ID: intellectual disability; NAAR: National Alliance for Autism Research; NDAR: National Database for Autism Research; NHGRI: National Human Genetics Research Institute; NIH: National Institutes of Health; NIMH: National Institute of Mental Health; PGC: Psychiatric Genetics Consortium; PPVT: Peabody Picture Vocabulary Test; RBS-R: Repetitive Behavior Scale-Revised; SNP: single-nucleotide polymorphism; SNV: single nucleotide variation; SRS: Social Responsiveness Scale; SSC: Simons Simplex Collection; TASC: The Autism Simplex Collection; VABS: Vineland Adaptive Behavioral Scales; WES: whole exome sequencing.

## Competing interest

The authors declare that they have no competing interests.

## Authors’ contributions

JDB: conception and design; data collection; analysis; manuscript writing; critical revision; final approval of manuscript; overall study coordination; financial support. NB: overall study coordination. JMB: overall study coordination. RJLA: data collection; analysis; manuscript writing; critical revision; final approval of manuscript. PB: Patrick Bender is now deceased and therefore unable to read and approve the final version of the manuscript. RB: data collection. EHC: conception and design; data collection; analysis; manuscript writing; critical revision; final approval of manuscript; overall study coordination. HC: conception and design; data collection; analysis; final approval of manuscript; overall study coordination. MC: conception and design; data collection. CMF: data collection; critical revision; final approval of manuscript; overall study coordination. JH: conception and design. DG: final approval of manuscript; financial support. SMK: data collection; critical revision; final approval of manuscript; overall study coordination. JIN: data collection; critical revision; final approval of manuscript. GO: conception and design; data collection. DP: conception and design; analysis; manuscript writing; final approval of manuscript; overall study coordination; financial support. FP: data collection; critical revision; final approval of manuscript; overall study coordination. SWS: conception and design; data collection; final approval of manuscript; overall study coordination; financial support. AS: conception and design; financial support. JSS: conception and design; data collection; analysis; manuscript writing; final approval of manuscript; overall study coordination; financial support. PS: data collection; final approval of manuscript. AMV: data collection; final approval of manuscript; overall study coordination. VV: conception and design; manuscript writing; final approval of manuscript; overall study coordination. LG: conception and design; data collection; analysis; manuscript writing; critical revision; overall study coordination; financial support. DG: data collection. AK: data collection; final approval of manuscript. LS: data collection; final approval of manuscript. AT: data collection. SB: data collection. GH: data collection. ML-S: data collection. FL: data collection. JMG: data collection. PC: data collection. SJG: data collection; final approval of manuscript. WMcM: overall study coordination. JM: data collection; analysis. JG: conception and design. MP-V: conception and design. ED: data collection. SS: data collection. CJMcD: data collection. DJP: data collection. JA: conception and design; data collection. GSC: data collection. JLH: conception and design; data collection. MP: data collection. ALN: data collection. CC: data collection. PF: conception and design. All authors read and approved the final manuscript.
